# A Multidisciplinary Approach to the Management of Severe Purpura Fulminans in a Burn Center: A Case Series

**DOI:** 10.7759/cureus.5478

**Published:** 2019-08-25

**Authors:** Mohammed Asif, Luis Quiroga, Tomer Lagziel, Seth B Ladd, Julie Caffrey

**Affiliations:** 1 Surgery, Burn Center, The Johns Hopkins University School of Medicine, Baltimore, USA; 2 Medicine, Burn Center, The Johns Hopkins University School of Medicine, Baltimore, USA; 3 General Surgery, St. John's Episcopal Hospital, Far Rockaway, USA

**Keywords:** purpura fulminans, sepsis, disseminated intravascular coagulation

## Abstract

Purpura fulminans is a life-threatening hematological emergency characterized by skin necrosis and disseminated intravascular coagulation requiring rapid diagnosis and treatment. We present a case series of patients with severe purpura fulminans who were managed via a multidisciplinary approach at a regional burn center. We report the burn unit perspective which includes current intensive care guidelines with early surgical intervention, in addition to a review of the pathology and clinical features of the disease. Proper wound management and expeditious surgical evaluation can help reduce the mortality and minimize amputations. Early referral to a burn center with a multidisciplinary team is recommended for the best outcomes in these patients.

## Introduction

Purpura fulminans (PF) is a rare but potentially fatal thrombotic disorder that can occur in association with hereditary or acquired deficiencies of the anticoagulants, protein C and S. The genetic form of the deficiency usually follows an autosomal dominant mode of inheritance and involves mutations to the PROC (Protein C) and PROS1 (Protein S1) genes [1]. Protein S deficiencies can also be observed in patients suffering from liver disease [2]. Most commonly, PF represents the progress from the acute inflammatory response to subsequent disseminated intravascular coagulation (DIC), associated with severe sepsis. PF provides a unique challenge to surgeons given its rapid cascade of events and high mortality rate. The mortality rate in PF patients is reported to be up to 50%, with the most common causes due to DIC and multi-organ failure [3].

We report a case series of patients with PF who were treated at a regional burn center. The purpose of this article is to highlight the clinical presentation and emphasize the importance of early aggressive supportive treatment and judicious use of surgical procedures to improve survival as well as functional outcomes [4].

## Case presentation

We performed a retrospective chart review of the patients admitted to our regional burn center from July 2015 to April 2019. Patients with diagnoses of purpura fulminans were identified. Their cases were reviewed to determine extent of the injury, surgical treatment, length of stay, complications, and outcomes.

Three cases of PF were identified (Table 1). The average length of stay was 47.33 days ranging from 30 to 69 days. The location of the injuries included bilateral upper and lower extremities. The average number of surgeries was 4, ranging from 3 to 5.

**Table 1:** Clinical profile, management and outcome of patients with purpura fulminans

Case	Age	Sex	Smoker	Presentation	Referral	Past Medical History	Organism	Affected Site	Adjunct Therapy	Surgical Procedure	Outcome
1	61	Female	No	Septic shock	Transfer from general surgery	Multiple myeloma and acute diverticulitis	Escherichia coli, Proteus mirabilis, Staphylococcus coagulase negative	Right flank and right lower extremity	Ventilation, inotropes, vasopressors, IV fluids, anticoagulants	Multiple surgical debridement, allografting and finally autografting	Survived
2	34	Male	Yes	Septic shock	Transfer from emergency department	Stab wounds, diaphragmatic repair and splenectomy (Not followed up with prophylactic vaccinations)	Streptococcal pneumoniae	Both upper and lower extremities	Ventilation, inotropes, vasopressors, IV fluids, IV antibiotics, haemodialysis, anticoagulants	Multiple surgical debridement, allograft, and finally below elbow amputation, bilateral below knee amputation	Survived
3	41	Male	No	Septic shock	Transfer from emergency department	Tuberculosis, acute pancreatitis, intravenous drug use, cellulitis	Streptococcal pyogenes	Left upper extremity and lower extremities	Ventilation, inotropes, vasopressors, IV fluids, IV antibiotics, anticoagulants	Multiple surgical debridement, allografting and autografting. Lower left extremity below-knee amputation	Survived

Case report 1

**Figure 1:**
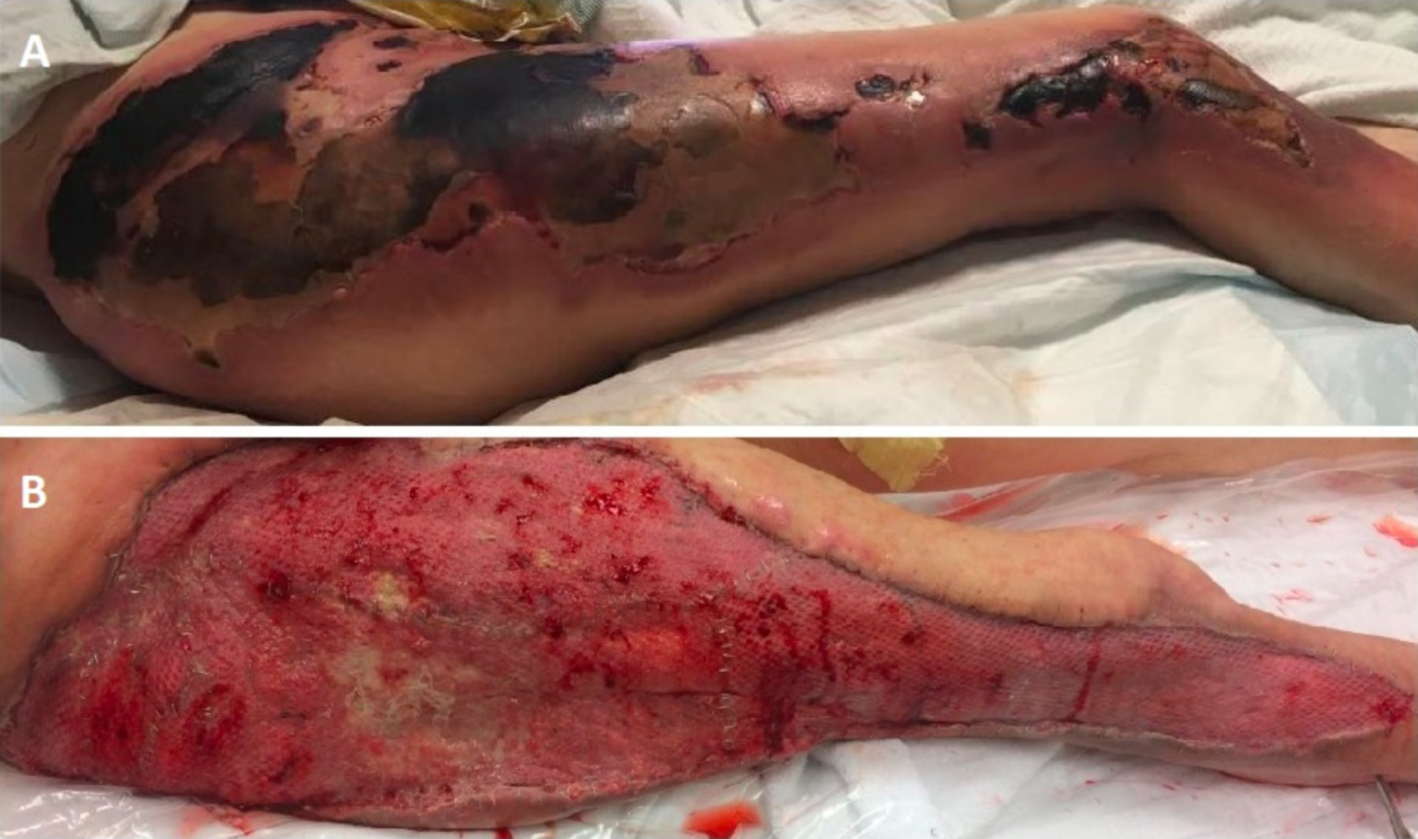
Injuries at initial presentation (A) and after definitive wound closure with split thickness skin grafting (B)

A 61-year-old female patient presented with a history of multiple myeloma with complete remission on thalidomide maintenance whose postoperative course complicated by an anastomotic leak and gram-negative septic shock after an elective colostomy reversal. Her blood cultures were positive for *Escherichia coli*, coagulase-negative Staphylococcus, and *Proteus mirabilis*. The patient returned to the operating room for source control, washout and placement of a diverting loop ileostomy. During this time, she developed hemorrhagic, necrotic skin of the right hip, thigh and lower leg leading to a diagnosis of PF (Figure 1A).

She was managed conservatively with complex wound care involving soaked dressings. After demarcation, debridement was performed on hospital day 27. She required multiple wound excisions, and negative pressure therapy wound dressing. She underwent definitive wound closure with split thickness skin grafting on hospital day 41 (Figure 1B).

Case report 2

A 34-year-old male smoker with a past medical history of emergent exploratory laparotomy and splenectomy after stab wound to the chest and abdomen presented with acute onset of high fevers, chills, and myalgia over the course of 24 hours. His initial laboratory was remarkable for leukocytosis, lactic acidosis to 12.5, new onset thrombocytopenia (from 262 k to 36 k), and increased levels of plasma d-dimers (D-dimer > 30.00). His clinical exam was notable for cold and pale palms and feet (with intact pulses) and a petechial rash covering his face, hands and feet. He developed septic shock requiring support with mechanical ventilation, vasopressors, IV fluids, and hemodialysis. He was empirically started on vancomycin and cefepime and was found to be bacteremic with gram-positive cocci (*Streptococcus pneumoniae*) secondary to community-acquired pneumonia. Upon more careful review of his past medical history, he had never received post-splenectomy vaccinations.

After one week, once stabilized, he was transferred to the burn intensive care unit (BICU) from the ED for management of his wounds. He had lesions involving the face, ears, hands, thighs, oral and feet.

**Figure 2:**
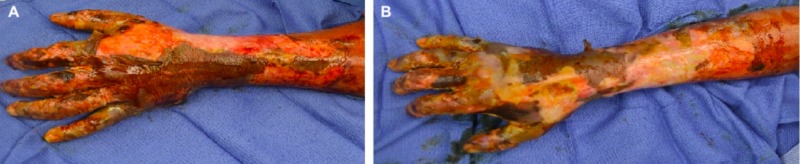
Injuries at the time of initial debridement on left hand (A) and right hand (B)

Initial attempts at limb salvage were made, however on hospital day 12, he underwent right distal forearm amputation to control the rapidly spreading infection, and excision and skin grafting to the left hand (Figure 2).

**Figure 3:**
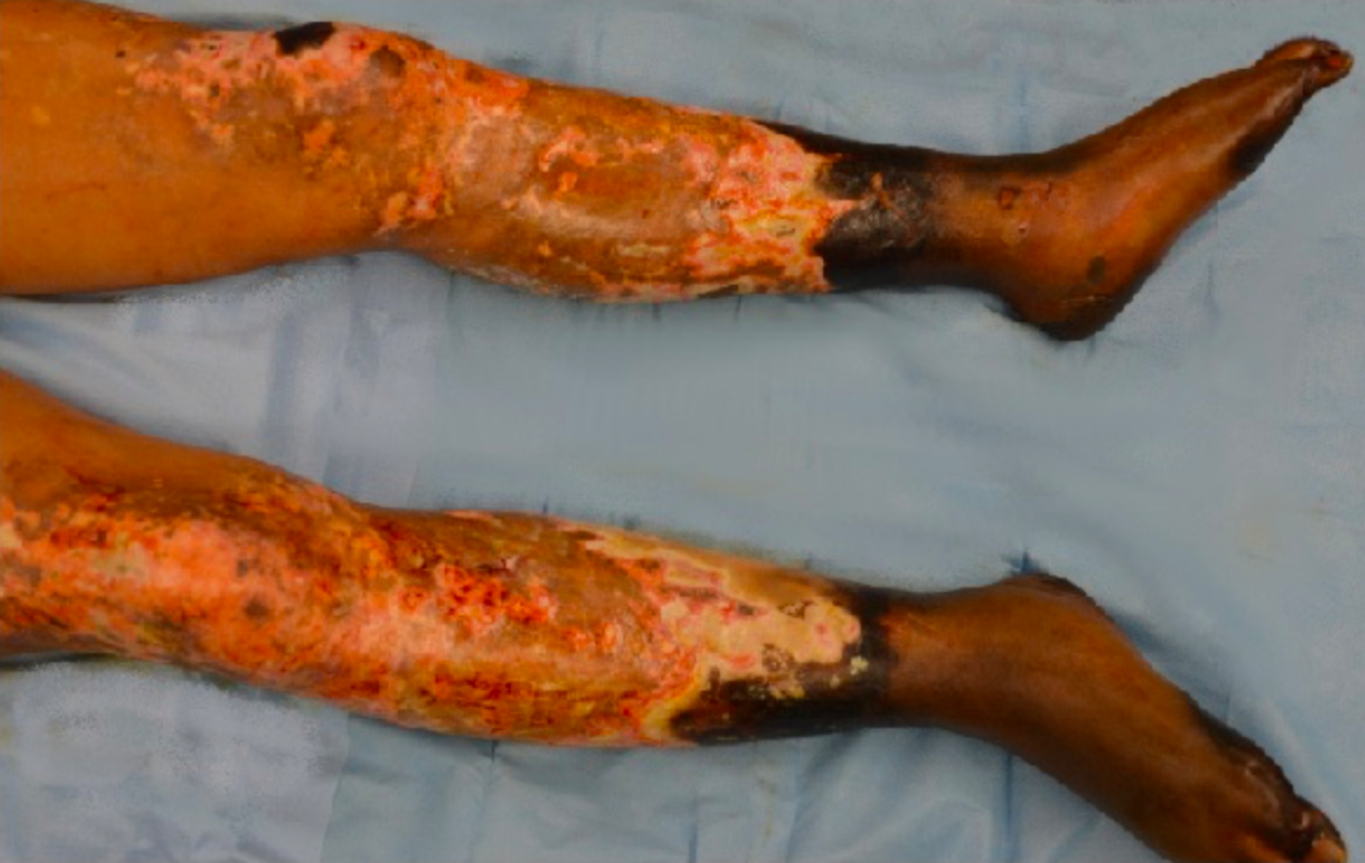
Lower extremity before initial debridement

On hospital day 19, the patient underwent bilateral below knee amputation (Figure 3).

One year later, the patient is able to walk with a prosthesis. He has some complaints of phantom limb.

Case report 3

A 41-year-old male smoker with a past medical history significant for tuberculosis treated in 2009, acute pancreatitis, intravenous drug use, recurrent episodes of cellulitis and subcutaneous abscesses secondary to skin popping presented to the emergency department (ED) with one-week history of worsening and intense left upper and lower extremity pain and swelling associated with fevers up to 40°C, weight loss, and hypotension. He was admitted to the Critical Care Unit with the diagnosis of sepsis secondary to soft tissue infection. On physical examination, he was found to have left upper extremity ulcers associated with a generalized erythematous rash. The initial laboratory workup was significant for leukocytosis, thrombocytopenia (18,000), hypofibrinogenemia, elevated PTT, and elevated PT consistent with disseminated intravascular coagulation. He developed septic shock complicated by acute kidney injury requiring hemodialysis. Blood cultures were positive for Group A Streptococcal (*Streptococcus pyogenes*) bacteremia.

**Figure 4:**
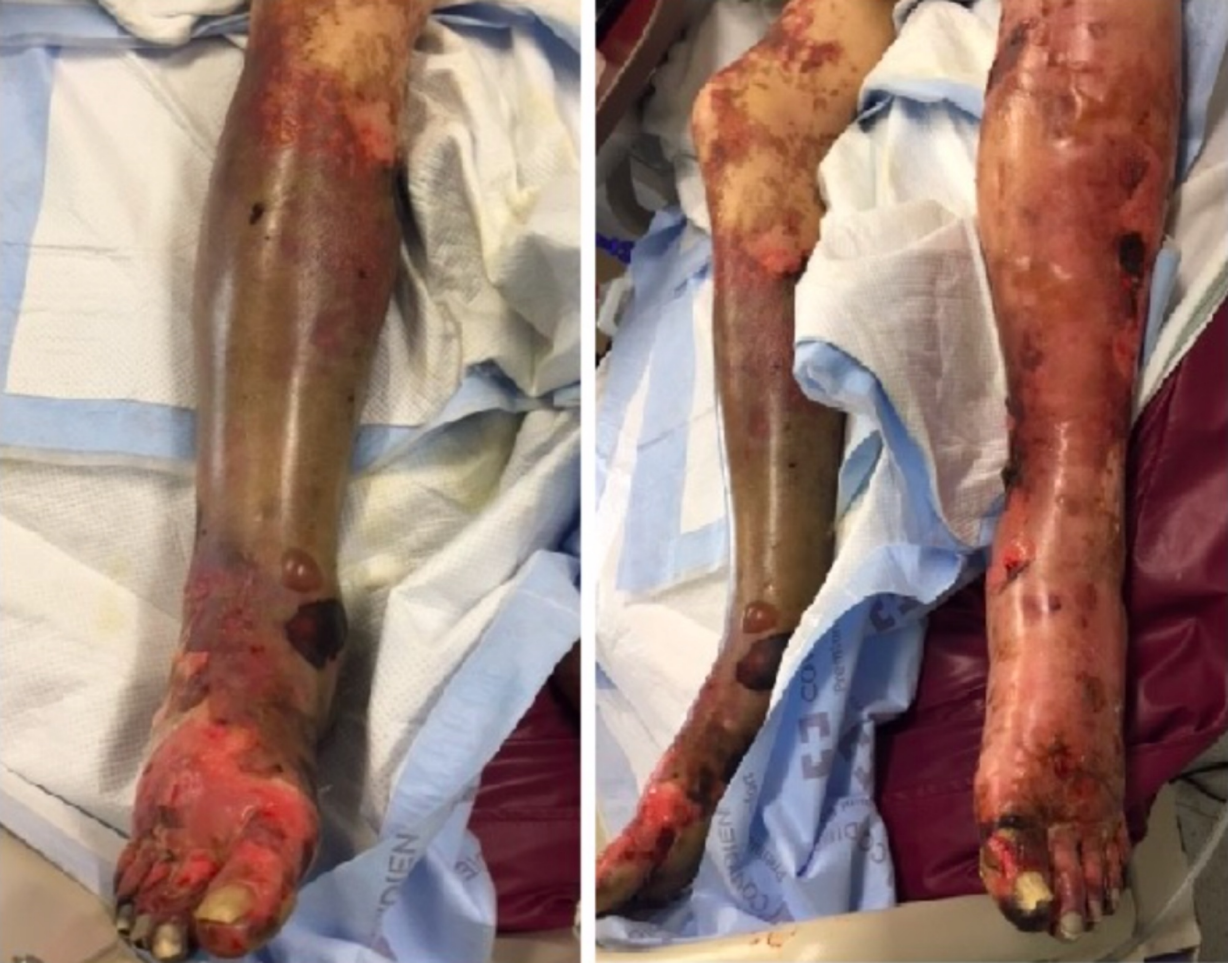
Lower extremities initial presentation

His ulcerated wounds progressed to reticulated confluent plaques involving the digits of his left hand extending to the rest of his left upper extremity. Also, plaques were noted on his inner thighs, nose, and ears. Similar findings were later observed in all the other extremities and part of his torso. Compartment syndrome was not observed in this patient as primary lower extremity pulses were palpable (femoral, popliteal, dorsalis pedis, posterior tibial). The patient was ultimately taken to the OR and underwent multiple excisions of his wounds to achieve control of the infection. His left upper extremity required split thickness skin graft that healed well. However, the left lower extremity did not recover well and he underwent left below knee amputation (Figure 4).

## Discussion

PF is an uncommon but severe and life-threatening thrombotic disorder [3]. PF requires a multidisciplinary treatment approach and early diagnosis represents a challenge and requires high suspicion [4]. Therefore, a differential diagnosis is an important initial step in assessing a patient. The following should be included: Henoch- Schonlein purpura (HSP), toxic epidermal necrolysis (TEN), allergic skin reaction and thrombocytopenic purpura (TP). HSP commonly presents in children following an upper respiratory tract infection with a symmetrical macular rash over the buttocks and posterior legs. This evolves into purpura within 24 hours; however, these are smaller, urticarial, are rarely associated with hemodynamic compromise, and seldom lead to necrosis [5]. TP is found in an afebrile patient and presents as a spontaneous non-palpable purpura and petechiae that does not lead to necrosis. Allergic skin reaction is erythematous in appearance and pruritic. The above can be excluded on clinical grounds and a combination of bacterial cultures and laboratory features can also be used to confirm PF.

The characteristic PF skin lesions initially begin as well-demarcated erythematous macules. This progresses rapidly to hemorrhagic necrosis and retiform purpura with vesicle or bulla formation resulting in gangrene [6]. These lesions mainly appear 12 to 24 hours after initial flu-like symptoms commence (fever, myalgia, and headache). Thrombosis-induced ischemia results in leakage of red blood cells and eventually skin necrosis [7]. The extremities are usually affected because of poor blood perfusion to distal tissues in the presence of septic shock [4,8].

The pathogenesis of sepsis-induced PF is not fully understood. Previous literature has suggested that the superantigen toxins produced by gram-positive bacteria disturb the coagulant activity of endothelial cells. This triggers the release of pro-inflammatory cytokines, resulting in toxic shock syndrome and the development of capillary thrombosis [9]. A deficiency of protein C in combination with this coagulation activation can result in high mortality [10]. Bacterial colonization of vessels in conjunction with this signaling pathway may explain purpuric lesions and the increase in vascular permeability, responsible for the severity of the shock [11].

Gram-positive bacteria and Gram-negative diplococci are common infectious agents. The most common presentation of PF in the setting of sepsis is associated with up to 20% *Neisseria meningitidis* cases, followed by *Streptococcus pneumoniae*, *Staphylococcus aureus*, and less frequently with groups A and B beta-hemolytic Streptococci,* Haemophilus influenza *and *Escherichia coli *[12], *Proteus mirabilis*, coagulase-negative Staphylococci, *Streptococcal pyogenes*, and *Streptococcal pneumoniae* were identified in our cases.

Transfer to a burn center with a multidisciplinary approach to treatment is ideal. Two of the three patients required amputation, and all recovered completely after excision and autografting. Early diagnosis and fasciotomy may help limb survival. Best attempts at limb salvage are necessary given the number of involved extremities. Rehabilitation is a long road for some of these patients and therefore multidisciplinary input involving occupational therapy, physiotherapy, dietitians, social work and pastoral care are required to transition these patients back into society and optimize functional outcome.

Initial conservative management of the wounds helps to minimize tissue loss and amputations. The infection is controlled by applying silver sulfadiazine (SSD) cream, acetic acid, clorpactin, and Vashe Wound Cleanser. This approach gives the wound bed time to clear and bacterial load reduce and can slow the development of severe DIC, in line with previous studies [13].

These cases highlight the importance of a multidisciplinary team management and an awareness of its clinical presentation to ensure effective support and better outcomes. Initially, early diagnosis and intensive care support are beneficial, followed by surgical evaluation and management. The aim is to remove necrotic skin, increase limb salvage and prevent loss of limb function and subsequent amputation [3,14]. Given the large area of body surface involved, management is best achieved in burn units where specialized nursing needs are provided alongside intensivists. Limb loss is common due to the high incidence of compartment syndrome combined with the extensive soft tissue loss secondary to purpura and limb ischemia [4]. Previous literature has suggested that delay in referral for intervention compounded with missed extremity compartment syndrome can also lead to significant tissue loss in PF patients. Prosthetic fitting and composite tissue allotransplantation will play an important role in PF survivor reconstruction in the future [13].

PF is a life-threatening condition often requiring major amputation. Early recognition, diagnosis, and subsequent treatment is crucial in preventing a fatality. It is possible that if the patients from these cases were admitted to the hospital earlier and stabilized quickly, their morbidities might have been less severe and overall outcomes would be more favorable. Autoamputation is of consideration when there is a dry gangrene component. Unfortunately, our patient in Case 2 had wet gangrene of the wounds which required aggressive debridement and subsequent amputation due to the overwhelming infection. An extensive review of the literature indicates that a regional burn center is the most suitable place to treat these patients and a multidisciplinary team can help optimize morbidity and mortality outcomes [15].

## Conclusions

We have presented these cases also to stress the importance of the underlying disease that may result in such a serious medical emergency. Where possible, supportive therapy in the intensive care unit should be a conservative first-line approach where native tissue can be preserved. However, an aggressive approach with excision, debridement, skin grafts and early amputation of ischemic limbs can also reduce the morbidity and mortality of PF cases. Surgeons must be mindful of the balance between removing necrotic tissue and the retention of as much digit functionality as possible.
